# Anemonefish use sialic acid metabolism as Trojan horse to avoid giant sea anemone stinging

**DOI:** 10.1186/s12915-025-02144-8

**Published:** 2025-02-15

**Authors:** Natacha Roux, Clément Delannoy, Shin-Yi Yu, Saori Miura, Lilian Carlu, Laurence Besseau, Takahiro Nakagawa, Chihiro Sato, Ken Kitajima, Yann Guerardel, Vincent Laudet

**Affiliations:** 1 Computational Neuroethology Unit, Okinawa Institute of Science and Technology, 1919-1 Tancha, Onna-Son, Okinawa, 904-0495 Japan; 2 Université de Lille, CNRS, UMR 8576 – UGSF - Unité de Glycobiologie Structurale et Fonctionnelle, Lille, 59000 France; 3 Marine Eco-Evo-Devo Unit, Okinawa Institute of Science and Technology, 1919-1 Tancha, Onna-son, Okinawa, Japan; 4 Sorbonne Université, CNRS, Biologie Intégrative des Organismes Marins, BIOM, Observatoire océanologique de Banyuls-sur-Mer, Banyuls-sur-Mer, 66650 France; 5 CNRS IRL 2028, Eco-Evo-Devo of Coral Reef Fish Life Cycle” (EARLY), Banyuls-Sur-Mer, France; 6 Institute for Glyco-Core Research (iGCORE), Nagoya University, Chikusa, Nagoya, 4648601 Japan; 7 Institute for Glyco-Core Research (iGCORE), Gifu University, Gifu, Japan; 8 Marine Research Station, Institute of Cellular and Organismic Biology (ICOB), Academia Sinica, 23-10, Dah-Uen Rd, Jiau Shi, I-Lan 262, Taipei, Taiwan

**Keywords:** Anemonefish, Sialic acid, Giant sea anemone

## Abstract

**Background:**

Anemonefish association with sea anemones is a prime example of mutualistic symbiosis. These fish live inside the sea anemone, benefitting from the protection of its toxic nematocysts, and in return, protect the anemone from its own predators. How anemonefish manage to avoid their host toxic stings remains unclear. One hypothesis suggests that low levels of sialic acids in anemonefish mucus prevent nematocyst discharge.

**Results:**

This study verified four predictions: (i) anemonefish mucus has lower sialic acid levels than non-symbiotic damselfish; (ii) this reduction is specific to mucus; (iii) during development, sialic acid levels inversely correlate with protection; (iv) sea anemone mucus has minimal sialic acids.

**Conclusions:**

We conclude that anemonefish regulates the level of sialic acids in their mucus to avoid nematocyst discharge. We also highlight several genes implicated in sialic acid removal that could explain the protection mechanisms in place. This mechanism, potentially used by *Dascyllus trimaculatus* juveniles, suggests a convergent strategy for mutualistic associations with sea anemones.

**Supplementary Information:**

The online version contains supplementary material available at 10.1186/s12915-025-02144-8.

## Background

Symbiosis, the intimate long-term association between two or more organisms of different species, is a fascinating biological phenomenon. Such an association can be beneficial to both partners (mutualism), to only one of them (commensalism), or even be detrimental to one partner (parasitism) [1]. One of the most striking examples is the long-term association between anemonefishes and their giant sea anemone hosts [2]. The 28 species of the *Amphiprion* genus that belong to the pomacentrids family have in common the ability to form social groups living in a close association with 10 sea anemones which belong to 3 distinct clades [2–4].

Studied since the end of the nineteenth century [5], this symbiosis is considered as a mutualistic relationship as the sea anemone provides a protection to the anemonefish thanks to their deadly tentacles whereas, by their aggressive behavior, the anemonefishes repel sea anemone predators. This symbiosis has always fascinated scientists for three main reasons. First, the anemonefishes can live safely inside the tentacles of their host otherwise known to discharge stinging nematocysts contained in a cnidocytes [6]. Nematocysts, which are threads releasing a cocktail of neurotoxins once it penetrates its target, are released after a combination of chemical and mechanical stimulation occurring at the surface of the cnidocytes [7–9]. Briefly, the release of sea anemone nematocysts requires first the fixation of specific substrates on chemoreceptors located at the surface of the tentacles. This will then cause structural modifications of the cnidocyte complex making them more sensitive to mechanical stimulation caused by swimming prey entering in contact with the tentacles (see [10] for review). It is clear that anemonefish are influencing somehow the triggering of these events as they succeed in living within the sea anemone tentacles. Second, there is a complex species specificity of this mutualistic relationship since a few anemonefish species in the wild live only in one sea anemone species (called specialists, e.g., *Amphiprion frenatus*, *A. sebae*, *A. biaculeatus*), whereas other may have between 2 or even 10 possible hosts (called generalists, e.g., *A. ocellaris*, *A. bicinctus*, *A. perideraion*, *A clarkii*) [4, 11, 12]. Third, this symbiosis is in fact a tripartite association as the giant sea anemones are themselves symbiotic animals that host a symbiotic dinoflagellate algae *Symbiodinium* providing them 80% of their energy via photosynthesis. It has also been shown that the 3 partners are metabolically connected [13, 14]. Indeed, anemonefish provide nitrogen and carbon to the host and its endosymbiotic zooxanthellae, playing therefore an important role in sea anemone nutrition [15, 16].

Even though numerous studies have tried to better understand the resistance of anemonefishes to sea anemone stinging, this question remains unresolved [2, 14]. Three main hypotheses have been proposed: (i) anemonefish have a thicker mucus layer than other fishes that protect them as a shield; (ii) anemonefish molecularly mimics the composition of anemone mucus; and (iii) anemonefish mucus lacks the trigger for firing sea anemone nematocysts. Individual evidence supports each of these claims. For example, it has been shown that *A. clarkii* mucus was three to four times thicker than that of other coral reef fish species, and did not elicit any response from the sea anemone [17]. Concerning the second hypothesis, it is proposed that anemonefish, by covering themselves with sea anemone mucus, would inhibit nematocyst discharge via a similar mechanism used by sea anemone to prevent nematocysts firing on their own tentacles [18]. Comparison of anemonefish mucus and sea anemone mucus revealed, for example, the presence of anemone antigens in *A. clarkii* mucus when inhabiting inside its host [19]. It has also been shown that anemonefish and sea anemone microbiome converge after association, providing an argument in favor of this hypothesis and also suggests the potential for microbial proteins to be involved in molecular mimicry [20, 21]. Arguments in favor of the third hypothesis come from genomic analysis that identified genes under positive selection at the base of the anemonefish radiation [22]. Some of these genes are implicated in sugar biogenesis, suggesting that difference in mucus composition may have been instrumental for the protection. This is supported by the observation that a sugar, the 5-*N*-acetylneuraminic acid (Neu5Ac), can stimulate cyclic adenosine monophosphate (cAMP) production and activate calcium channels in sea anemone tentacles, hinting at a role of Neu5Ac in chemo-sensitization of nematocyst discharge [8]. In accordance with these observations, it has been shown that *A. ocellaris* mucus lacks Neu5Ac [23]. These data suggest that the lack of Neu5Ac may play a key role in avoidance of nematocyst discharge. However, the reports listed above are somewhat anecdotical and do not provide compelling evidence regarding the mechanism that allows anemonefish, in contrast to other fishes, to live unharmed among anemone tentacles.

Neu5Ac belongs to a class of acidic monosaccharides called sialic acids that are themselves a subset of a family of α-keto acid monosaccharides with a 9-carbon backbone called nonulosonic acids (NulOs). Neu5Ac typically modifies glycolipids and protein glycans (abundant in vertebrates) by substituting the extremity of their associated glycan moieties. As a result, sialic acids are the most external monosaccharides and serve as mediators of numerous biological processes such as ligand-receptor and cell–cell interactions [24]. Although Neu5Ac is among the most abundant sialic acid encountered in nature, more than 50 other forms of sialic acids have been identified among which 5-*N*-glycolylneuraminic acid (Neu5Gc) and 2-keto-3-deoxy-nononic acid (Kdn), which are well represented in microorganisms and most animals (such as echinoderms, mammals, and teleost fish) [25–27]. It should be noted that Neu5Ac, Neu5Gc, and Kdn have all been identified in numerous species of fishes, although sometimes in an organ-specific manner [28–31]. It is thus highly possible that a precise regulation of the types of sialic acids present in the fish skin mucus could explain the inability of anemonefish to trigger their host stinging cells.

If sialic acids play a substantial role in anemonefish protection, we propose the following four predictions: (i) sialic acid concentrations in the mucus of anemonefish species and a sequentially symbiotic damselfish should be substantially smaller than in the mucus of non-symbiotic damselfishes (i.e., the lineage most closely related to anemonefish) which are known to trigger sea anemone nematocyst discharge; (ii) as sialic acids are essential for many biological process, we would not expect to see their levels affected in other organs; (iii) during anemonefish larval development, we should observe a concurrent shift in both the level of sensitivity towards sea anemone stinging and the concentration of sialic acid in the mucus. Indeed, young larvae have been suspected to trigger nematocyst discharge, they should contain more sialic acid than juveniles or adults; (iv) if anemonefish are using a Trojan horse strategy, that is, if the absence of sialic acid prevents nematocyst discharge, we could expect low levels of sialic acids in sea anemone mucus. In this study, we test and validate these predictions, and we therefore propose that the specific absence of sialic acids on fish mucus indeed explains why anemonefish live unharmed among their host tentacles.

## Results

### Prediction 1: mucus sialic acid composition of anemonefishes is different compared to damselfish mucus

Here we conduct analyses of sialic acid composition in anemonefishes and damselfishes, both in specimens maintained in husbandry (at the marine station of Banyuls-sur-Mer and at the Okinawa Institute of Science and Technology) and in wild caught fishes (in French Polynesia and in Okinawa).

We analyzed the mucus of 5 anemonefish species maintained in laboratory without sea anemone (*A. biaculeatus*, *A. clarkii*, *A. frenatus*, *A. ocellaris*, and *A. percula*, *n* = 3 per species) and juveniles of two damselfish species (*Dascyllus trimaculatus* and the non-symbiotic species *Acanthochromis polyacanthus*, *n* = 9 per species). The interest of *Dascyllus trimaculatus* is that this damselfish species is known to live associated with sea anemone when accepted by dominant anemonefish at the juvenile stage whereas adults are mostly found over small patches of reef and rubble areas, or hiding inside corals branches [32, 33]. Occasionally, adults can be found close to sea anemones [34]. They could represent an interesting case of convergence since, while being part of damselfish, they are not directly related to anemonefish [35].

**Fig. 1:**
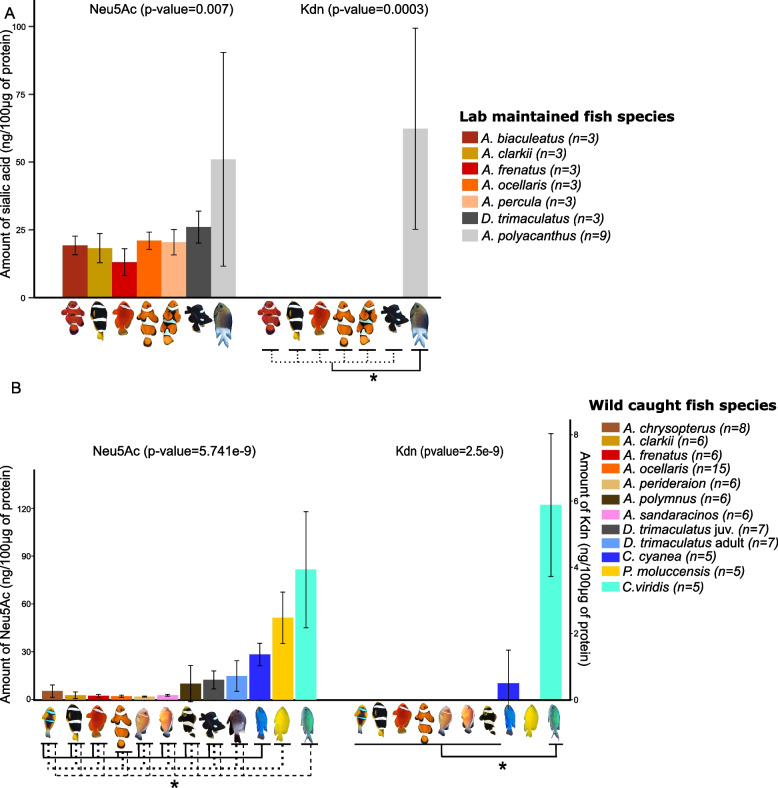
Sialic acid levels in lab and wild caught pomacentridae showing less Neu5Ac in symbiotic species. **A** Neu5Ac and Kdn levels (expressed in ng/100 µg of protein) in lab reared fish (held without sea anemone): five symbiotic anemonefish species (*Amphiprion biaculeatus*, *A. clarkii*, *A. frenatus*, *A. ocellaris*, and *A. percula*) and 1 symbiotic (when juvenile) damselfish species (*Dascyllus trimaculatus*) compared to one non-symbiotic damselfish species (*Acanthochromis polyacanthus*). A non-parametric Kruskal–Wallis test (*p* value on top of the graph) was performed. **B** Neu5Ac and Kdn levels (expressed in ng/100 µg of protein) in wild caught fish: seven symbiotic anemonefish species (*A. chrysopterus*, *A. clarkii*, *A. frenatus*, *A. ocellaris*, *A. perideraion*, *A. polymnus*, and *A. sandaracinos*), one symbiotic damselfish species (D. trimaculatus at the juvenile and adult stage), and three non-symbiotic species (*Chrysiptera cyanea*, *Pomacentrus moluccensis*, and *Chromis viridis*). A non-parametric Kruskal–Wallis test (*p* value on top of the graph) followed by a pairwise Wilcoxon rank sum test was performed for Neu5Ac. Only significant differences between anemonefish species, *D. trimaculatus*, and each damselfish species are displayed for Neu5Ac and indicated by a star (*). A non-parametric Kruskal–Wallis test was performed for the Kdn levels, followed by a pairwise Wilcoxon rank sum test (*p* value on top of the graph) (significant differences are displayed by a star (*) below the graph). Data are presented in Additional file 6: Table S1

Our analyses indicate that the mucus of symbiotic species contains less sialic acids than the non-symbiotic species (Fig. 1A). More specifically, we observe a significant effect of species on sialic acid levels (Neu5Ac *p* value = 0.007, Kdn *p* value = 0.0003). Both anemonefish and juvenile *D. trimaculatus* mucus have less Neu5Ac than *A. polyacanthus* and the deaminated sialic acid Kdn is not detected in anemonefish and juvenile *D. trimaculatus* whereas it is detected in *A. polyacanthus* (Fig. 1A). Neu5Gc was not detected in any of the analyzed species.

As anemonefish maintained in laboratory and used in this study were not associated with sea anemone, we performed the same analyses with the mucus from individuals sampled in the wild, comparing 7 anemonefish species of the genus *Amphiprion*: *A. chrysopterus* from French Polynesia (*n* = 8) as well as *A. clarkii* (*n* = 6), *A. frenatus* (*n* = 6), *A. ocellaris* (*n* = 15), *A. perideraion* (*n* = 6), *A. polymnus* (*n* = 6), *A. sandaracinos* (*n* = 6), one symbiotic damselfish species (*D. trimaculatus* juveniles and adults, *n* = 6 per stage), and 3 non-symbiotic damselfish, *C. cyanea* (*n* = 5), *P. moluccensis* (*n* = 5), and *C. viridis* (*n* = 5), all from Okinawa. The results of these analyses (Fig. 1B) confirmed our results obtained with lab-maintained fish (Fig. 1A).

Symbiotic anemonefish species and symbiotic damselfish *D. trimaculatus* showed significantly less Neu5Ac compared to the three non-symbiotic damselfish species (mean anemonefish Neu5Ac levels comprised between 2.6 and 9.9 ng/100 µg of protein, mean *D. trimaculatus* Neu5Ac levels comprised between 12.3 and 14.7 ng/100 µg of protein whereas mean damselfish Neu5Ac levels were comprised between 28.2 and 81.5 ng/100 µg of protein; *p* value < 0.05). As previously mentioned for husbandry fishes, no Neu5Gc (data not shown) nor Kdn were detected in anemonefish mucus (Fig. 1B). Neu5Gc was also undetected in the three damselfish, but Kdn was detected in various proportions in *C. viridis* and *C. cyanea* but not in *P. moluccensis* (Fig. 1B). Interestingly, when we compared the amount of sialic acid present in fish maintained in laboratory (without sea anemone) from those living in the wild (with sea anemone), we observed a statistically significant greater amount in the mucus of specimens not associated with sea anemone (Additional file 1: Fig. S1A). In order to determine if this difference was linked to the presence of sea anemone with fish sampled in the wild, we measured Neu5Ac levels in *A. ocellaris* individuals living in aquaria with a sea anemone for 3 months and compared with fish held without sea anemone in the same conditions (Additional file 1: Fig. S1B). No difference was observed between the two conditions (with/without sea anemone) suggesting that the sea anemone has no impact on the regulation of Neu5Ac in *A. ocellaris* mucus and that the difference observed between lab and wild sampled fish is likely due to other environmental parameters.

Taken together these results validate the first prediction: sialic acid levels (Neu5Ac and Kdn taken together) are lower in anemonefish and *D. trimaculatus* (associated with giant sea anemone at juvenile stage) than in non-symbiotic damselfish whatever the conditions or the origins of the fish.

### Prediction 2: anemonefish display reduced sialic acid content only in mucus compared to other organs

**Fig. 2:**
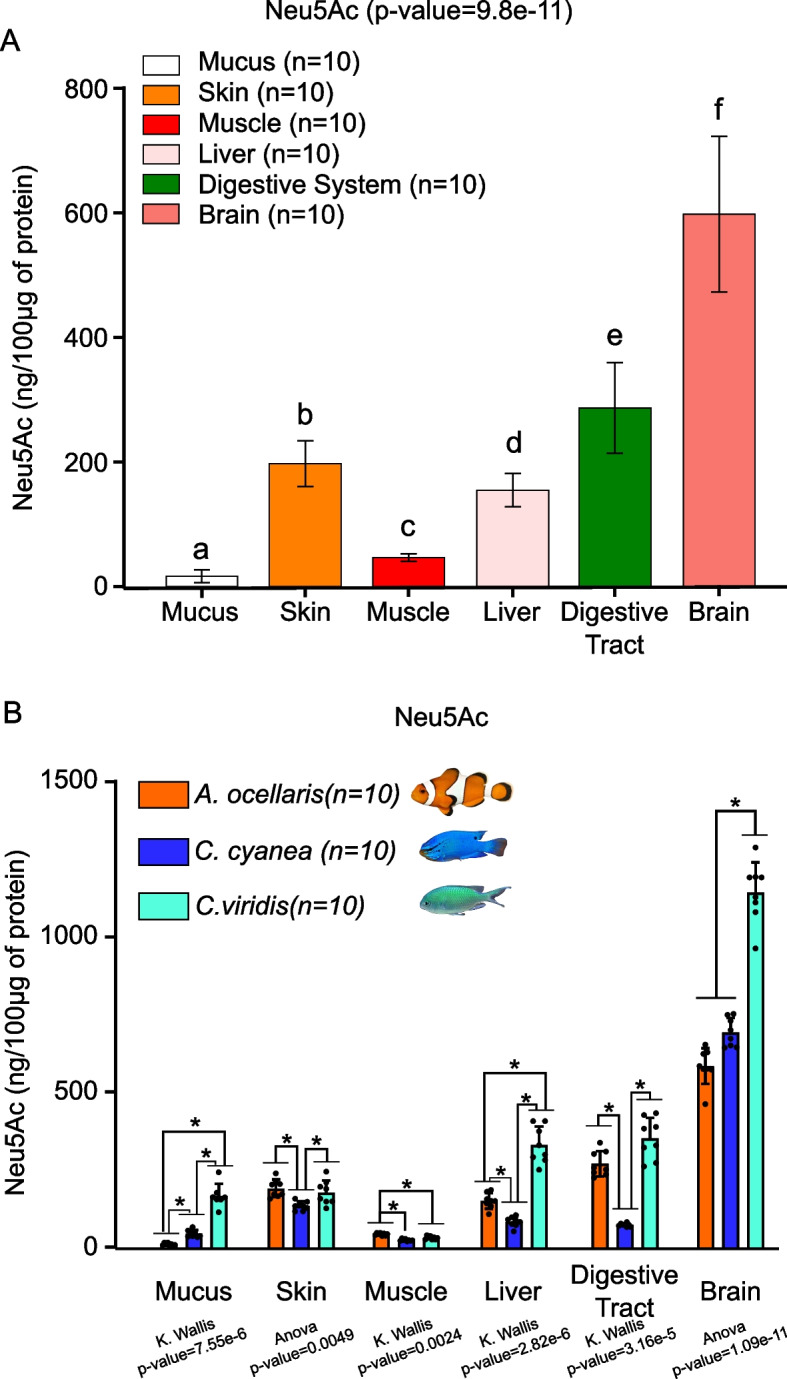
Sialic acid levels in pomacentrids various organs confirm a decrease specific to *A. ocellaris* mucus. **A** Neu5Ac levels in *A. ocellaris* organs (mucus, skin, muscle, liver, digestive tract, and brain). A non-parametric Kruskal–Wallis test (*p* value = 9.8e − 11), followed by a pairwise Wilcoxon rank-sum test, was performed to compare Neu5Ac levels between *A. ocellaris* organs. Organs displaying different letter are significantly different. **B** Neu5Ac levels in *A. ocellaris*, *C. cyanea*, and *C. viridis* organs (mucus, skin, muscle, liver, digestive tract, and brain). Depending on the organ, a one-way ANOVA or a non-parametric Kruskal–Wallis test was performed, followed by a Tukey HSD or a pairwise Wilcoxon rank-sum test to compare Neu5Ac levels between the three species for each organ. Significant differences are displayed by a star (*). Data are presented in Additional file 6: Table S2

To test the second prediction, we measured sialic acid levels in various organs in comparison to mucus of the symbiotic species *A. ocellaris* (*n* = 10, which were never in contact with sea anemone) compared to two non-symbiotic species (*C. cyanea* and *C. viridis*, *n* = 10 per species). Interestingly, the results revealed much higher levels of Neu5Ac in all anemonefish organs compared to the mucus, suggesting that the observed reduction is specific to mucus (Fig. 2A, *p* value < 0.05). When compared to damselfish organs, *A. ocellaris* Neu5Ac levels were not necessarily lower in all organs. For example, Neu5Ac was significantly higher in *A. ocellaris* skin, muscle, liver, and digestive tract compared to *C. cyanea* (Fig. 2B, *p* value < 0.05). On the contrary, *C. viridis* Neu5Ac levels were significantly higher than both *A. ocellaris* and *C. cyanea* in liver and brain (Fig. 2B, *p* value < 0.05). Kdn was only detected in *C. viridis* mucus, liver, and digestive tract, but not in any organs of *A. ocellaris* or *C. cyanea* (Additional file 1: Fig. S1B).

These results support the second prediction: sialic acid levels are specifically decreased in anemonefish mucus but not in other organs, including skin, which demonstrates that the expression of sialic acids is regulated in tissues and organs to fulfill their functions. The fact that skin sialic acid level is not different between anemonefish and *C. viridis* has interesting implications that will be discussed later.

### Prediction 3: resistance towards stinging is acquired during metamorphosis and correlates with sialic acid content

**Fig. 3:**
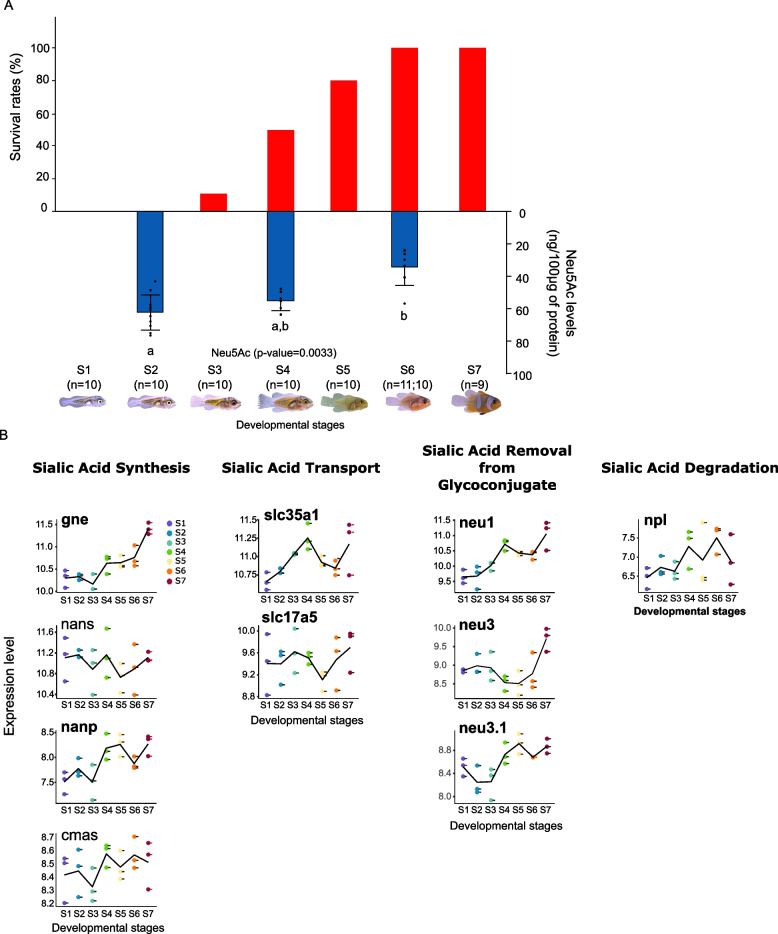
Survival rates and sialic acid production changes during *A. ocellaris* larval development. **A** Combined graphs showing in red the survival rates (percentage) of *A. ocellaris* larvae sampled at different developmental stages [37] and in blue Neu5Ac levels (expressed in ng/100 µg of protein) of larvae sampled before metamorphosis (stage 2) and during metamorphosis (stage 4 and 6). A one-way ANOVA (*p* value displayed on the graph) followed by a Tukey HSD test was performed on Neu5Ac levels to compare each developmental stage. Stage with different letter has significantly different Neu5Ac levels. **B** Expression levels of genes involved in sialic acid synthesis (*gne, nans, nanp, cmas*), transport (*slc35a1, slc17a5*), removal from glycoconjugate (*neu1, neu3, neu3.1*), and degradation (*npl*). Genes written in bold are significantly differentially expressed between pre-metamorphosis stage (S1 and/or S2 and/or S3) and metamorphosis stages (S5 and/or S6 and/or S7) (see Roux et al. [37] for the description of gene expression analysis method). Data are presented in Additional file 6: Tables S3 and S4

Although it has always been claimed that young anemonefish larvae are sensitive to sea anemone tentacles, no clear evidence has been brought for confirmation. To unequivocally determine when clownfish starts to become resistant towards sea anemone stinging, *Amphiprion ocellaris* larvae were sampled at each of the 7 developmental stages (previously described in Roux et al. [36]), put in contact with the giant sea anemone *S. gigantea* and survival rates were recorded for each stage. We observed that young larvae (stage 1 and 2) are extremely sensitive towards stinging as none survived after contact with sea anemone tentacles (Fig. 3A). Survival rates started to increase at stage 3 and 4 (10% and 50%, respectively, Fig. 3A) and reached 100% at stage 6 and 7. Stage 4 marks the onset of metamorphosis and the transition between the oceanic dispersal phase and the reef phase [36, 37]. After entering a reef, anemonefish larvae must locate a suitable sea anemone to settle [38]. It is thus necessary for them to be able to enter their host without being stung, which is what has been observed here. Once larvae start metamorphosing at stage 4, survival rates increase, demonstrating that they are ready to settle into a sea anemone.

In addition to assessing survival rates, we also measured the levels of sialic acid on stage 2, 4, and 6 larvae to determine if the survival rate increase might corroborate with a change in the sialic acid composition of the larvae. Among the three sialic acids commonly measured (Neu5Ac, Neu5Gc, and Kdn), only Neu5Ac was detected, and we observed a significant decrease at stage 6 compared to stage 2 and 4 (*p *value < 0.05, Fig. 3B). It is worth noting that, because of the size of clownfish larvae, mucus collection was not possible, therefore we compared the sialic acid content in the whole animal. Despite this experimental limitation, these results could be interpreted as confirmation of the third prediction.

These data suggest that the resistance is acquired during metamorphosis. As the transcriptomic changes occurring during anemonefish larval development and metamorphosis have been previously analyzed [37], we scrutinized the expression of sialic acid metabolism genes during metamorphosis to eventually extend the correlation between protection and sialic acids. For this, transcriptomic data obtained by Roux et al. [37] for each developmental stages (bulk RNA sequencing on entire larvae) were used to retrieve the normalized expression levels of 33 genes involved in Neu5Ac metabolism. These genes were categorized into five genes sets: Neu5Ac synthesis, Neu5Ac transport, Neu5Ac degradation, Neu5Ac removal from glycans as well as Neu5Ac transfer and fixation on glycans. The complete pathway and the expression pattern of each gene are presented in Additional files 2 and 3 (Figs. S2 and S3). We observed quite a heterogenous change of expression, which is not surprising since, because of the small size of the larvae, these transcriptomic data were obtained from entire fish in which sialic acids have important roles in various organs (as shown in Fig. 2). It is therefore not surprising to see such tight regulation of key metabolic genes during larval development. However, as exemplified in Fig. 3B, an increased expression in early juvenile stages is observed for three genes encoding for enzymes implicated in sialic acid removal called neuraminidase (namely *neu1*, *neu3*, and *neu3.1*, Fig. 3B). The gene encoding for neuraminidase 1 (*neu1*) peaks at stage 4 and later increases in juveniles is particularly interesting as it cleaves sialic acid from substrate such as glycoproteins and could therefore be used to remove sialic acid from proteins expressed by mucous cells. The two genes *neu3* and *neu3.1* have similar expression patterns, with strong increases until stage 7 and could also be implicated in similar activities. This may indicate that the removal of sialic acid is an active phenomenon that is established during metamorphosis, ensuring that the juvenile emanating from metamorphosis can enter safely in a sea anemone (see “Discussion”).

### Prediction 4: low level of sialic acids is sea anemone mucus

The fourth prediction suggests that anemonefish might actually utilize the same mechanism as sea anemones to prevent nematocyst discharge from being triggered against their own tentacles. Nematocyst discharge, as well as toxin synthesis, has a metabolic cost for sea anemone [39–41] and it is likely that these organisms have developed a system not to sting themselves. One relevant possibility would be that giant sea anemones also use the lack of sialic acid to avoid stinging themselves and in that view, anemonefish may in fact behave as Trojan horse high jacking the very same strategy to avoid triggering nematocyst discharge. This would imply that giant sea anemone would also have low levels of sialic acid. For this reason, we compared sialic acid levels in four giant sea anemones (*Heteractis magnifica*, *Stichodactyla gigantea*, *Heteractis crispa*, and *Entacmaea quadricolor*) that represents the three main clades of giant sea anemone associated to anemonefish [3].

We did not observe significant amounts of Neu5Ac, Neu5Gc, Kdn, or any other type of sialic acid in the mucus sampled from these four species. Some signals corresponding to potential sialic acids were detected but their intensity was below the detection threshold (Additional file 4: Fig. S4). These data therefore confirm the fourth prediction, suggesting that the absence of sialic acid in sea anemone mucus may be linked to their protection against their own nematocysts and that anemonefish use this system as a Trojan horse to avoid being stung.

## Discussion

Known since the nineteenth century [5], the symbiotic relationship between giant sea anemone and anemonefish still holds secrets for the scientific community who is eager to understand how these fish are able to live unharmed in their deadly host. Our study brings new elements to this question and reinforces the hypothesis that the lack of sialic acids, and more specifically Neu5Ac, avoid anemonefish to trigger nematocyst discharge of their host.

### Sialic acid levels negatively correlate anemonefish sensitivity towards sea anemone stinging

A novel finding of our study is that the ability of anemonefish to live safely in the sea anemone tentacles is acquired during the larval development. Like most of the marine fish, anemonefish life cycle is composed of an oceanic larval dispersal followed by a sedentary coastal phase. They reproduce in the vicinity of their sea anemone host, laying eggs on a substrate close to the sea anemone. Right after hatching, larvae are transported into the open ocean and are not supposed to enter in contact with the sea anemone. However, to the best of our knowledge, no studies have directly investigated if the anemonefish larvae were innately resistant to their host stinging. Interestingly, we demonstrate that the decrease of Neu5Ac levels is negatively correlated with the increase of survival rates of *A. ocellaris* larvae. Our results indeed clearly show that the newly hatched larvae of *A. ocellaris* are highly sensitive to sea anemone stinging as survival rates are low at the beginning of the development. However, survival rates reached 100% at the end of the larval development. The fact that Neu5Ac levels follow a reverse tendency compared to survival rates and decreased during larval development clearly suggests that sensitivity towards sea anemone stinging might be linked to Neu5Ac levels.

### Anemonefish specifically decrease sialic acid levels in their mucus

By comparing the levels of sialic acids between 9 species of anemonefish (*A. biaculeatus*, *A. clarkii*, *A. frenatus*, *A. ocellaris*, *A. percula*, *A. chrysopterus*, *A. perideraion*, *A. polymnus*, and *A. sandaracinos*), one species of symbiotic damselfish (*Dascyllus trimaculatus*), as well as 4 non-symbiotic damselfish (*Acanthochromis polyacanthus*, *Chrysiptera cyanea*, *Pomacentrus moluccensis*, and *Chromis viridis*) sampled either in a lab environment or in the wild, we also corroborated and extend the results obtained by Abdullah and Saad [23]: anemonefish and symbiotic damselfish *D. trimaculatus* have less sialic acids than non-symbiotic damselfish species. In addition, in accordance with the fact that sialic acids have important biological functions [24], we observe that this decreased level is tissue specific and affects only the mucus of anemonefish.

### Anemonefish use sialic acid metabolism as a Trojan horse to get protection from sea anemone tentacles

The analysis of the sialic acid composition of the mucus isolated from sea anemones did not allow us to observe significant amounts of Neu5Ac, Neu5Gc, Kdn, or any other type of sialic acid (Additional file 4: Fig. S4). Although some signals corresponding to potential sialic acids were observed, their intensity was below the detection threshold that would allow them to be distinguished from background noise. These experimental results are not in accordance with what has been obtained by Abdullah and Saad [23] who detected high amounts of sialic acids. However, the analysis method used in their study, based on thiobarbituric acid, is not specific to sialic acids and is known to be reactive with other compounds like RNA and oxidized lipids. On the contrary, the method used in our study is highly specific to sialic acid detection; we are confident that the data obtained on sea anemones are in accordance with the fact that sialic acids have never been so far conclusively detected in another species of cnidarians (from our knowledge). Further investigation should be carried on investigating the presence of genes involved in sialic acid metabolism in cnidaria to validate our results.

Nematocyst discharge (and toxin synthesis) has a metabolic cost for sea anemone [39–41]. It is thus very likely that these organisms have developed a system to not sting themselves as proposed originally by Schlichter who suggested that sea anemone mucus contains inhibitory substances that prevent self-stimulation and nematocyst discharge, and that anemonefishes acquire these substances during acclimation [18, 42]. Based on the results obtained in our study, the model would be different in that it suggests that sea anemones have low levels of Neu5Ac in their mucus to avoid auto-stinging and that anemonefish highjacked this system to enter safely into their host. Interestingly, this model could explain why anemonefish can live unharmed in other cnidarians as frequently observed in captivity [32].

### What could be the mechanisms at play?

How the anemonefish decreases the amount of sialic acid in their mucus is still unclear and may result from the combination of several mechanisms. First, it has been shown previously that among genes positively selected at the base of the anemonefish radiation and therefore potentially involved in the symbiosis establishment, two encodes for proteins with a functional link with N-acetylated sugars: versican core protein (*vcan*) and the O-GlcNAc transferase (*ogt*) [22]. Versican core protein is known to be a critical extracellular matrix regulator of immunity and inflammation [43] that interacts with several matrix molecules including glycosaminoglycans containing N-acetylhexosamine [44]. Expression of versican core protein in clownfish skin is thought to bind to N-acetylated sugars and could therefore mask them to the host chemoreceptors therefore preventing nematocyst discharge. Two *vcan* genes were identified in our transcriptomic data and one, *vcana*, who is expressed in the epidermis, showed an increase from stage 4 (marking the onset of metamorphosis, Additional file 5: Fig. S5) suggesting it may play a role in this key period where the young fish acquire the resistance [22, 37]. On the other hand, protein O-GlcNAcase has the potential to cleave N-acetylated sugars from different cell surface molecules [45] and has also been found to be expressed in anemonefish epidermis [22]. One *ogt* gene has been identified in the transcriptomic data set used in this study (*ogt1*) and its expression level decreased from stage 3 in whole fish (Additional file 5: Fig. S5) but we still do not know how its expression is regulated in skin during metamorphosis.

A second possible mechanism could be the direct tissue specific regulation of genes implicated in sialic acid biochemistry that is sialyltransferases that transfer sialic acid to nascent oligosaccharide, but also neuraminidases (also called sialidase) that remove sialic acids from glycoconjugates. Despite coming from entire individuals and therefore not representative of the exquisite tissue-specific regulation probably at play, the expression levels provide some interesting hints that can be explored in further studies. Indeed, we observed an activation of the pathways governing removal of Neu5Ac exemplified by an increased expression of *neu1*, *neu3*, and *neu3.1* coinciding with metamorphosis, the increase of survival rates, and the decrease of Neu5Ac levels. Further experiments are required to address gene expression in a tissue specific manner to understand the regulation mechanism involved in the reduction of sialic acid levels in anemonefish.

Another relevant hypothesis that could explain the local regulation of sialic acid content in mucus could be the action of bacteria involved in sialic acid removal. Indeed, several bacteria are known to possess enzymes (sialidases and neuraminidases) involved in sialic acid removal [46] and it has been shown that the mucus of fish and sea anemone converge after contact in terms of microbiota [20, 21]. This is an interesting possibility as it would explain why in many cases naive anemonefish need to acclimate to their sea anemone and would be also consistent with the model suggesting a chemical mimicry of anemonefish with sea anemone mucus. An interesting path to follow would be therefore to test if host bacteria are able to remove sialic acids from fish mucus.

### Other possible mechanism at play

Our data suggest that the low level of sialic acid plays an important role in the protection mechanism that allows anemonefish to live unharmed inside sea anemone tentacles. It would indeed be challenging to conceive how anemonefish can thrive and reproduce over numerous generations in tentacles that still daily sting and harm them, even at minimal levels. The specific lack of sialic acid in their mucus allows them to avoid such a situation. However, we must clarify that we do not claim that this lack of sialic acid is the only mechanism at play. Indeed, this does not explain why some anemonefishes need to acclimate for several minutes before entering in a sea anemone, suggesting that a second process, likely associated to chemical mimicry, is at play. Recent results suggest that the sea anemone that has a direct interest to host anemonefish because of territorial defense and metabolic exchange may also play a role in allowing anemonefish to settle by discharging fewer nematocysts at familiar anemonefish after delayed mucus adaptation [47]. The anemonefish-giant sea anemone relationships appear much more profound and complex than anticipated and we believe this includes the mechanisms to protect each other in their long-term association.

It is also known that nematocysts can fire following chemical stimulation (like the presence of sialic acid) but also via the activation of mechanoreceptors. It is thus very likely that other unknown mechanisms also participate to the protection. For example, there may be difference in the organization of the epidermis between anemonefish and damselfish such as epidermis thickness, scale organization and thickness, and amount of mucus that allow the anemonefish to be better protected against low nematocyst discharge that would normally harm or even kill another fish. In other words, it is likely that the lack of sialic acid in the mucus is necessary but is coupled to additional mechanism.

Another important aspect that is not explained by our results is the specificity of association between the 28 species of anemonefish and the ca. 10 species of giant sea anemone. It is clear that there are complex and still largely unknown rules of association with some species of anemonefish being specialists some other being generalist [14]. In our study, we also did not note any difference in sialic acid levels when considering anemonefish host specificity. For example, the generalist *A. clarkii* showed similar levels of Neu5Ac when compared to the specialist species *A. frenatus* (2.69 ± 2.03 (sd) ng/100 µg of protein, 2.31 ± 0.98 (sd) ng/100 µg). Therefore, the model we tested in this study does not explain these effects that are likely due in part to ecological preference, chemoattraction mechanisms but that could also be linked to a sensitivity of some species to the toxic compounds released by the sea anemone [14, 48].

### Is this a general phenomenon?

One intriguing result of our study is the fact that both juvenile and adult domino damselfish (*Dascyllus trimaculatus*) also contain low levels of sialic acid in their mucus (Fig. 1A, B). This species is known to live associated with sea anemone at juvenile stage when accepted by dominant anemonefish [33]. Adults can also be found from time to time close to sea anemones and are known to be unharmed by sea anemone tentacles but they mostly live above small patches of reef and rubble areas [32, 34]. As domino damselfish is not closely related to anemonefish [35], this clearly suggests a case of convergence and reinforces the association between the lack of sialic acid and the prevention of nematocyst release.

In this context, it is interesting to note that several species of fish, such as cardinalfishes (Apogonidae), wrasses (Labridae), hawkfishes (Cirrhitidae), butterflyfishes (Chaetodontidae), a scaled blenny (Clinidae), and even a temperate greenling (Hexagrammidae), also live loosely associated with sea anemones [34, 49, 50]. Many of those are in fact sensitive to nematocysts and can have lesions after contact with the tentacles. In other cases, such as the labrisomid *Starksia hassi* or the cardinalfish *Apogon moluccensis*, no apparent lesions are observed despite full contact with the tentacles suggesting that once again a mechanism avoiding nematocyst discharge exists. It is also worth to note that many invertebrates such as shrimps or crabs are living permanently inside sea anemone, as anemonefish [6, 51]. Another interesting case, while not being a symbiosis, is the case of nudibranch that feed on cnidarians as they must defend themselves from the prey’s nematocysts and it has been shown that their mucus inhibit the discharge of nematocysts from sea anemone tentacles [52]. It will be very interesting to study these cases to see if the convergence observed between anemonefish and the non-closely related damselfish *D. trimaculatus* extends to other species, vertebrates and invertebrates.

## Conclusions

In conclusion, our study provides compelling evidence supporting the hypothesis that anemonefish actively reduce the levels of sialic acids in their mucus as a protective mechanism against nematocyst discharge from their giant sea anemone hosts. Remarkably, our observations also suggest a shared utilization of these mechanisms by unrelated damselfish juveniles, underscoring the broader ecological significance of convergent adaptations in facilitating mutualistic interactions within marine ecosystems. Overall, our research offers valuable insights into the intricate mechanisms of the relationships between unrelated organisms and opens avenues for further exploration in symbiosis biology.

## Methods

### Mucus sampling

A total of seven species were sampled in Banyuls-sur-Mer Marine station husbandry: five anemonefishes (*A. biaculeatus n* = 3, *Amphiprion ocellaris n* = 3, *A. percula n* = 3, *A. clarkii n* = 3, *A. frenatus n* = 3), one damselfish inhabiting sea anemone at juvenile stage (*Dascyllus trimaculatus n* = 3), and one non-symbiotic damselfish (*Acanthochromis polyacanthus n* = 9). Each species was maintained in closed recirculatory system filled with artificial sea water (Red Sea salt, Antinéa, France) and without sea anemones. Temperature was maintained at 26°C, salinity at 34 g/L, and a 14/10 h light/dark photoperiod was applied.

A total of ten species were sampled in the wild: one species in Moorea, French Polynesia (*A. chrysopterus n* = 8), five species in Okinawa Island, Japan (*A. clarkii n* = 6, *A. frenatus n* = 6, *A. ocellaris n* = 15, *A. perideraion n* = 6, *A. polymnus n* = 6, *A. sandaracinos n* = 6), one symbiotic damselfish species (*Dascyllus trimaculatus* at juvenile and adult stage, *n* = 7 per stage) and three non-symbiotic damselfish species, also encountered in Okinawa (*Chrysiptera cyanea n* = 5, *Pomacentrus moluccensis n* = 5, *Chromis viridis n* = 5). Fish were sampled whether by snorkeling or diving using hand nets.

Mucus collection was always conducted using the following protocol with sterile material and gloves to avoid contamination of the samples. Fish were individually anesthetized in MS222 (200 mg/L, Sigma Aldrich) and transferred in a glass petri dish without water. Sterile cell scraper (SARSTEDT, Nümbrecht, Germany) was used to gently scrap each flank of the fish from gills to tail (5 times per side without touching the gills). The fish were then gently placed back in a container filled with sea water for awakening and place back in their aquarium or released in the wild. Mucus was washed off from cell scraper and petri dish with 2 ml of ultrapure water, transferred in a glass tube and kept at − 20°C until extraction and analysis. Gloves were changed between species to avoid any contamination.

### Organ sampling

To determine if sialic acid composition in anemonefish is decreased in the mucus or in the entire body, fish maintained in aquaria without sea anemone were dissected and the following organs were sampled: mucus, skin, muscle, liver, digestive tract, and brain. The anemonefish *A. ocellaris* as well as 2 non-symbiotic damselfish *C. cyanea* and *C. viridis* were sampled for comparison (*n* = 10 per species). *A. ocellaris* juveniles were obtained from OIST husbandry and juveniles of both *C. cyanea* and *C. viridis* were obtained from a local petshop (Makeman, Uruma city). Fish were euthanized in MS222 (400 mg/L) and transferred in a glass petri dish for mucus collection (described above) and organ dissection. Organs were stored separately in 1.5-ml Eppendorf tubes and kept at − 20°C until extraction and analysis. Dissection tools and petri dish were rinsed and disinfected with ethanol between each individual to avoid contamination between samples.

### Sialic acid hydrolysis and DMB derivatization

All samples were lyophilized before extraction. Once lyophilized, samples were incubated in CHAPS extraction buffer (8 M urea; 2% CHAPS, 50 mM DTT, 1X protease inhibitor) and maintained under constant agitation at 4°C overnight. Protein extracts were then centrifuged at 20,000 g, 4°C for 10 min and supernatants were collected. Protein concentration was determined by the Pierce™ BCA Protein Assay Kit—Reducing Agent Compatible, according to the manufacturer’s instructions. Forty micrograms of protein extract were loaded into preconditioned HTS 96-well plates with hydrophobic Immobilon-P PVDF membrane and incubated for 30 min at 37°C. The wells were washed 6 times with 200 µL mQ water prior to centrifugation (1 min, 500 g). Sialic acids attached to glycoconjugates were released at 60°C for 3 h in 0.1 M trifluoroacetic acid. Released sialic acids were collected by centrifugation (1 min, 1000 g) and lyophilized. They were then subsequently coupled to 1,2-diamino-4,5-methylenedioxybenzene dihydrochloride (DMB). Samples were heated at 50°C for 2 h in the dark in 7 mM DMB, 1 M β-mercaptoethanol, and 18 mM sodium hydrosulfite in 5 mM acetic acid. Sialic acids coupled to DMB (DMB-Sia) were then analyzed by liquid chromatography fluorescence detector (LC-FLD).

### Quantitation analysis of DMB-Sia on LC-FLD

DMB-labeled sialic acids were injected into the Prominence LC-20AB micro LC system (Shimadzu). Samples were applied to an analytical LC column (InfinityLab Poroshell 120 EC-C18, 4.6 × 150 mm, 2.7 µm) and separated isocratically by a solvent mixture of acetonitrile/methanol/water (9:7:84) and identified by referring to the elution positions of standard Neu5Ac, Neu5Gc, and Kdn derivatives. Individual sialic acid derivatives were quantified by integration of fluorescence signals after HPLC separation, plotted against standard curves of corresponding authentic standards.

### Survival experiment

To determine at which stage anemonefish larvae become resistant to sea anemone stinging, larvae of *A. ocellaris* were sampled at each developmental stage (7 distinct stages according to the developmental table of Roux et al. [36]) and put individually using a transparent pipette in contact with the tentacles of the sea anemone *Stichodactyla gigantea* (stage 1 to 5: *n* = 10, stage 6: *n* = 11, stage 7: *n* = 9). Individuals stuck to tentacles and unable to escape were counted as dead and individuals able to freely swim in between the tentacles without sticking to them were counted as surviving. Total number of dead individuals was then used to determine the survival rates for each developmental stage. Larvae were raised in a closed system using natural filtered sea water following methods described in Roux et al. [53].

### Developmental stage sampling

To assess the sialic acid composition and quantity during *A. ocellaris* larval development, three developmental stages were sampled according to the developmental table of Roux et al. [36]: before metamorphosis (stage 2 *n* = 10), beginning of metamorphosis (stage 4 *n* = 10), and during metamorphosis (stage 6 *n* = 10). Samples were frozen and processed as described above.

### Sea anemone mucus sampling

Four giant sea anemones (*Heteractis magnifica*, *Stichodactyla gigantea*, *Heteractis crispa*, and *Entacmaea quadricolor*) that represents the three main types of giant sea anemone associated to anemonefish [3]. Animals were collected from the wild in Okinawa and maintained in a natural sea water open circuit in OIST marine station. Each specimen was gently caught with a bucket filled with sea water from its own tank and gently brought to the surface to emerge tentacles. Mucus was then collected by putting a glass petri below some tentacles and gently scrapped with a cell scraper. Mucus was then collected and handled similarly as fish mucus sample. Collection was repeated 4 times on different tentacles for each species.

### *A. ocellaris* with/without sea anemone mucus sampling

Two groups of 7 *A. ocellaris* were placed in separate aquariums at OIST marine station. One group was placed with a sea anemone (*S. gigantea*) and one group was held alone. Mucus sampling occurred as described above 3 months after *A. ocellaris* individuals were in contact with their host.

### Sialic acid signaling gene pathway expression level

Expression levels of 33 genes involved in sialic acid pathway were retrieved from each *A. ocellaris* larval developmental stages (*n* = 3 larvae per stage) using the transcriptomic data set published in Roux et al. and Salis et al. [37, 54, 55]. Those genes were categorized into the following 5 sets. Genes encoding for enzymes involved in Neu5Ac synthesis: *gne* (UDP-GlcNAc 2-epimerase/ManNAc kinase), *nans* (Neu5Ac 9-phosphate synthase), *nanp* (Neu5Ac 9-phosphate phosphatase), *cmas* (CMP-Neu5Ac synthetase). Genes encoding for enzymes involved in Neu5Ac transport: *slc35a1* (CMP-Sia transporter), *slc17a5* (sialin). Genes encoding for enzymes involved in Neu5Ac degradation: *npl* (N-acetylneuraminate pyruvate lyase). Genes encoding enzymes involved in the transfer and fixation of Neu5Ac on sialoglycans (glycoproteins, glycolipids, glycoRNA): sialyltransferases. Finally, genes encoding enzymes involved in sialic acid removal from sialoglycans: *neu1*, *neu3*, and *neu3.1* (neuraminidase 1, 3, and 3.1).

### Statistical analysis

Sialic acid levels were analyzed using RStudio software [56]. When testing the effects of several groups on sialic acid levels, one-way ANOVA was performed for Neu5Ac followed by a Tukey HSD test for post hoc comparison when parametric test could be used or a non-parametric Kruskal–Wallis test followed by a pairwise Wilcoxon rank sum test was performed with Benjamini and Hochberg correction. When testing the effects of only 2 groups on sialic acid levels, a parametric test of Student or a non-parametric Wilcoxon-Mann–Whitney test was performed.

## Supplementary Information

Additional file 1: Fig. S1 A) Comparison of Neu5Ac levels between lab maintained anemonefish species and wild caught anemonefish species (*Amphiprion clarkii*, *Amphiprion frenatus*, *Amphiprion ocellaris*). A parametric test of Student or a non-parametric Wilcoxon-Mann–Whitney test was performed to compare the mean Neu5Ac levels between lab and wild environment for each species. Significant differences are displayed by a star (*) for each species. B) Comparison of Neu5Ac levels between *A. ocellaris* held without and with sea anemone (*S. gigantea*) in aquarium at Okinawa marine station. A non-parametric Wilcoxon-Mann–Whitney test was performed but no significant difference was observed between the two groups. C) Levels of Kdn detected in organs of *Amphiprion ocellaris*, *Chrysiptera cyanea*, and *Chromis viridis*. Data are presented in Additional file 6: Table S1.

Additional file 2: Fig. S2 Complete signaling pathway of Neu5Ac metabolism associated with gene expression levels retrieved from transcriptomic data obtained by Roux et al. and Salis et al. [37, 54, 55]. Green genes are involved Neu5Ac synthesis, red genes in Neu5Ac degradation, blue genes in Neu5Ac removal from sialoglycans, and gray genes in Neu5Ac transport. Genes written in bold are significantly differentially expressed between pre metamorphosis stage (S1 and/or S2 and/or S3) and metamorphosis stages (S5 and/or S6 and/or S7) (see Roux et al. [1] for the description of gene expression analysis method). Data are presented in Additional file 6: Table S4.

Additional file 3: Fig. S3 Expression levels of genes encoding enzymes, called sialyltransferase, involved in the transfer and fixation of Neu5Ac on sialoglycans. Expression levels were classified into 4 categories (increase, surge at stage 4 or 5, decrease, and relatively stable). Data are presented in Additional file 6: Table S4.

Additional file 4: Fig. S4 Neu5Ac levels in *Heteractis magnifica*, *Stichodactyla gigantea*, *Heteractis crispa*, and *Entacmaea quadricolor* below the detection limit. Data are presented in Additional file 6: Table S5.

Additional file 5: Fig. S5 Expression levels of genes encoding proteins with a functional link with N-acetylated sugars: versican core protein (*vcan*) and the O-GlcNAc transferase (*ogt*) [22]. Versican core protein is known to be a critical extracellular matrix regulator of immunity and inflammation [43] that interacts with several matrix molecules including glycosaminoglycans containing N-acetylhexosamine [44] (see Roux et al. [37] for the description of gene expression analysis method). Data are presented in Additional file 6: Table S4.

Additional file 6: Tables S1–S5. Tables displaying all the data allowing to generate the figures and supplementary figures presented in this manuscript. Table S1 Sialic acid levels in anemonefish and damselfish mucus; Neu5Ac levels in anemonefish maintained with or without sea anemone. Table S2 Sialic acid levels measured in *Amphiprion ocellaris*, *Chrysiptera cyanea*, and *Chromis viridis* organs (brain, muscle, skin, liver, digestive tract, mucus). Table S3 Survival rates of *Amphiprion ocellaris* after contact with sea anemone tentacles and Neu5Ac levels measured in *A. ocellaris* larval stages. Table S4 Expression levels of genes involved in Neu5Ac pathways or having a functional link with N-acetylated sugars measured during *A. ocellaris* larval development using transcriptomic analysis. Table S5 Neu5Ac levels measured in sea anemone mucus.

## Data Availability

All data generated or analysed during this study are included in this published article, its supplementary information files and publicly available repositories. Transcriptomic data used in this paper are available under the following reference PRJNA482393 in NCBI (https://www.ncbi.nlm.nih.gov/bioproject/PRJNA482393/).

## Abbreviations

Neu5Ac: 5-N-acetylneuraminic acid
cAMP: Cyclic adenosine monophosphate
NulOs: Nonulosonic acids
Neu5Gc: 5-N-glycolylneuraminic acid
Kdn: 2-Keto-3-deoxy-nononic acid
gne: UDP-GlcNAc 2-epimerase/ManNAc kinase
nans: Neu5Ac 9-phosphate synthase
nanp: Neu5Ac 9-phosphate phosphatase
cmas: CMP-Neu5Ac synthetase
slc35a1: CMP-Sia transporter
slc17a5: Sialin
neu1, neu3, neu3.1: Neuraminidase
npl: N-acetylneuraminate pyruvate lyase
vcan: Versican core protein
ogt: O-GlcNAc transferase
DMB: 1,2-Diamino-4,5-methylenedioxybenzene dihydrochloride
LC-FLD: Liquid chromatography fluorescence detector
OIST: Okinawa Institute of Science and Technology

## References

[1] Dimijan G. Evolving together: the biology of symbiosis, part 1. BUMC Proc. 2000;13(3):217. PMC1317043 16389385

[2] da Silva KB, Nedosyko A. Sea anemones and anemonefish: a match made in heaven. In: Goffredo S, Dubinsky Z, éditeurs. The cnidaria, past, present and future: the world of Medusa and her sisters. Cham: Springer International Publishing; 2016. p. 425‑38. 10.1007/978-3-319-31305-4_27.

[3] Kashimoto R, Tanimoto M, Miura S, Satoh N, Laudet V, Khalturin K. Transcriptomes of giant sea anemones from Okinawa as a tool for understanding their phylogeny and symbiotic relationships with anemonefish. Zoolog Sci. 2022;39(4):374–87. 10.2108/zs210111 35960028

[4] Kashimoto R, Mercader M, Zwahlen J, Miura S, Tanimoto M, Yanagi K, et al. Anemonefish are better taxonomists than humans. Curr Biol. 2024;34(5):R193–4. 38471445 10.1016/j.cub.2023.07.051

[5] Collingwood C. Note on the existence of gigantic sea-anemones in the China sea, containing within them quasi-parasitic fish. Ann Mag Nat Hist. 1868;4(1):31–3.

[6] Mebs D. Chemical biology of the mutualistic relationships of sea anemones with fish and crustaceans. Toxicon. 2009;54(8):1071–4. 19268681 10.1016/j.toxicon.2009.02.027

[7] Lotan A, Fishman L, Zlotkin E. Toxin compartmentation and delivery in the cnidaria: the nematocyst’s tubule as a multiheaded poisonous arrow. J Exp Zool. 1996;275(6):444–51. 8795288 10.1002/(SICI)1097-010X(19960815)275:6<444::AID-JEZ6>3.0.CO;2-O

[8] Ozacmak VH, Thorington GU, Fletcher WH, Hessinger DA. N-acetylneuraminic acid (NANA) stimulates in situ cyclic AMP production in tentacles of sea anemone (Aiptasia pallida): possible role in chemosensitization of nematocyst discharge. J Exp Biol. 2001;204(11):2011–20. 11441042 10.1242/jeb.204.11.2011

[9] Tardent P. The biology of nematocysts. Academic Press. San Diego; 1988. 309‑332 p.

[10] Anderson PAV, Bouchard C. The regulation of cnidocyte discharge. Toxicon. 2009;54(8):1046–53. 19268492 10.1016/j.toxicon.2009.02.023

[11] Fautin DG, Allen GR. Anemonefishes and their host sea anemones: a guide for aquarists and divers. Perth, WA: Western Australian Museum; 1997. p. 160.

[12] Litsios G, Pearman PB, Lanterbecq D, Tolou N, Salamin N. The radiation of the clownfishes has two geographical replicates. Bellwood D, éditeur. J Biogeogr. 2014;41(11):2140–9.

[13] Verde A, Cleveland A, Lee RW. Nutritional exchange in a tropical tripartite symbiosis II: direct evidence for the transfer of nutrients from host anemone and zooxanthellae to anemonefish. Mar Biol. 2015;162(12):2409–29.

[14] Hoepner CM, Fobert EK, Abbott CA, Silva KB da. No place like home: can omics uncover the secret behind the sea anemone and anemonefish symbiotic relationship? In: Evolution, development and ecology of anemonefishes. Ravasi T. et Laudet V. Boca Raton: CRC Press; 2022.

[15] Cleveland A, Verde EA, Lee RW. Nutritional exchange in a tropical tripartite symbiosis: direct evidence for the transfer of nutrients from anemonefish to host anemone and zooxanthellae. Mar Biol mars. 2011;158(3):589–602.

[16] Cui G, Liew YJ, Li Y, Kharbatia N, Zahran NI, Emwas AH, et al. Host-dependent nitrogen recycling as a mechanism of symbiont control in Aiptasia Krediet CJ, éditeur. PLOS Genet. 2019;15(6):e1008189. 31233506 10.1371/journal.pgen.1008189 PMC6611638

[17] Lubbock R. The clownfish/anemone symbiosis: a problem of cell recognition. Parasitology. 1981;82(159):173.

[18] Elliott JK, Mariscal RN, Roux KH. Do anemonefishes use molecular mimicry to avoid being stung by host anemones? J Exp Mar Biol Ecol. 1994;179(1):99–113.

[19] Elliott JK, Mariscal RN. Acclimation or innate protection of anemonefishes from sea anemones? Copeia. 1997;1997(2):284–9.

[20] Pratte ZA, Patin NV, McWhirt ME, Caughman AM, Parris DJ, Stewart FJ. Association with a sea anemone alters the skin microbiome of clownfish. Coral Reefs. 2018;37(4):1119–25.

[21] Roux N, Lami R, Salis P, Magré K, Romans P, Masanet P, et al. Sea anemone and clownfish microbiota diversity and variation during the initial steps of symbiosis. Sci Rep. 2019;9(1):19491. 31862916 10.1038/s41598-019-55756-w PMC6925283

[22] Marcionetti A, Rossier V, Roux N, Salis P, Laudet V, Salamin N. Insights into the genomics of clownfish adaptive radiation: genetic basis of the mutualism with sea anemones. Genome Biol Evol. 2019;11(3):869–82. 30830203 10.1093/gbe/evz042 PMC6430985

[23] Abdullah NS, Saad S. Rapid detecion of N-acetylneuraminic acid from false clownfish using HPLC-FLD for symbiosis to host sea anemone. Asian J Appl Sci. 2015;3(5):858–64.

[24] Schauer R. Sialic acids as regulators of molecular and cellular interactions. Curr Opin Struct Biol. 2009;19(5):507–14. 19699080 10.1016/j.sbi.2009.06.003 PMC7127376

[25] Angata T, Varki A. Chemical diversity in the sialic acids and related alpha-keto acids: an evolutionary perspective. Chem Rev. 2002;102(2):439–69. 11841250 10.1021/cr000407m

[26] Chen X, Varki A. Advances in the biology and chemistry of sialic acids. ACS Chem Biol. 2010;5(2):163–76. 20020717 10.1021/cb900266r PMC2825284

[27] Schauer R. Achievements and challenges of sialic acid research. Glycoconj J. 2000;17(7–9):485–99. 11421344 10.1023/A:1011062223612 PMC7087979

[28] Aoki K, Kumagai T, Ranzinger R, Bergmann C, Camus A, Tiemeyer M. Serum N-glycome diversity in teleost and chondrostrean fishes. Front Mol Biosci. 2021;8. https://www.frontiersin.org/articles/10.3389/fmolb.2021.778383. 10.3389/fmolb.2021.778383 PMC8631502 34859056

[29] Inoue S, Kanamori A, Kitajima K, Inoue Y. KDN-glycoprotein: a novel deaminated neuraminic acid-rich glycoprotein isolated from vitelline envelope of rainbow trout eggs. Biochem Biophys Res Commun. 1988;153(1):172–6. 3377785 10.1016/s0006-291x(88)81204-x

[30] Venkatakrishnan V, Padra JT, Sundh H, Sundell K, Jin C, Langeland M, et al. Exploring the Arctic charr intestinal glycome: evidence of increased N-glycolylneuraminic acid levels and changed host–pathogen interactions in response to inflammation. J Proteome Res. 2019;18(4):1760–73. 30848132 10.1021/acs.jproteome.8b00973

[31] Yamakawa N, Vanbeselaere J, Chang LY, Yu SY, Ducrocq L, Harduin-Lepers A, et al. Systems glycomics of adult zebrafish identifies organ-specific sialylation and glycosylation patterns. Nat Commun. 2018;9(1):4647. 30405127 10.1038/s41467-018-06950-3 PMC6220181

[32] Allen GR. Anemonefishes. New Jersey: Their classification and biology. Tropical Fish Hobbyist Publications. Neptune City; 1975. p. 288.

[33] Hayashi K, Tachihara K, Reimer JD. Anemonefish aggressiveness affects the presence of Dascyllus trimaculatus co-existing with host anemones. Mar Biol. 2020;167(6):84.

[34] Feeney WE, Brooker RM, Johnston LN, Gilbert JDJ, Besson M, Lecchini D, et al. Predation drives recurrent convergence of an interspecies mutualism. Ecol Lett. 2019;22(2):256–64. 30481409 10.1111/ele.13184

[35] Tang KL, Stiassny MLJ, Mayden RL, DeSalle R. Systematics of damselfishes. Ichthyol Herpetol. 2021;109(1):258–318.

[36] Roux N, Salis P, Lambert A, Logeux V, Soulat O, Romans P, et al. Staging and normal table of postembryonic development of the clownfish (Amphiprion ocellaris). Dev Dyn. 2019;248(7):545–68. 31070818 10.1002/dvdy.46 PMC6771578

[37] Roux N, Miura S, Dussenne M, Tara Y, Lee S hua, de Bernard S, et al. The multi-level regulation of clownfish metamorphosis by thyroid hormones. Cell Rep. 2023;42(7):112661. 37347665 10.1016/j.celrep.2023.112661

[38] Barth P, Berenshtein I, Besson M, Roux N, Parmentier E, Banaigs B, et al. From the ocean to a reef habitat: how do the larvae of coral reef fishes find their way home. VIE MILIEU-LIFE Environ. 2015;95(2):91–100.

[39] Fautin DG. Structural diversity, systematics, and evolution of cnidae. Toxicon Off J Int Soc Toxinology. 2009;54(8):1054–64. 10.1016/j.toxicon.2009.02.024 19268491

[40] Sachkova MY, Macrander J, Surm JM, Aharoni R, Menard-Harvey SS, Klock A, et al. Some like it hot: population-specific adaptations in venom production to abiotic stressors in a widely distributed cnidarian. BMC Biol. 2020;18(1):121. 32907568 10.1186/s12915-020-00855-8 PMC7488265

[41] Kaposi KL, Courtney RL, Seymour JE. Implications of bleaching on cnidarian venom ecology. Toxicon X. 2022;13:100094. 35146416 10.1016/j.toxcx.2022.100094 PMC8819380

[42] Schlichter D. Macromolecular mimicry: substances released by sea anemones and their role in the protection of anemone fishes. In: Mackie GO, éditeur. Coelenterate ecology and behavior. Boston, MA: Springer US; 1976. p. 433‑41. 10.1007/978-1-4757-9724-4_46.

[43] Wight TN, Kang I, Evanko SP, Harten IA, Chang MY, Pearce OMT, et al. Versican-a critical extracellular matrix regulator of immunity and inflammation. Front Immunol. 2020;11:512. 32265939 10.3389/fimmu.2020.00512 PMC7105702

[44] Wu YJ, La Pierre DP, Wu J, Yee AJ, Yang BB. The interaction of versican with its binding partners. Cell Res. 2005;15(7):483–94. 16045811 10.1038/sj.cr.7290318

[45] Bathina AR. Effect of substrate availability and O-GlcNAse inhibition on hyaluronan synthesis and intracellular trafficking of HAS3 in MV3 melanoma cells. Finland: UNIVERSITY OF EASTERN FINLAND, Faculty of Health Sciences, School of Pharmacy; 2014.

[46] Li Y, Chen X. Sialic acid metabolism and sialyltransferases: natural functions and applications. Appl Microbiol Biotechnol. 2012;94(4):887–905. 22526796 10.1007/s00253-012-4040-1 PMC3534974

[47] Hoepner CM, Fobert EK, Rudd D, Petersen O, Abbott CA. da Silva KB. Friend, food, or foe: sea anemones discharge fewer nematocysts at familiar anemonefish after delayed mucus adaptation bioRxiv. 2024;02(22):581653.

[48] Nedosyko AM, Young JE, Edwards JW, da Silva KB. Searching for a toxic key to unlock the mystery of anemonefish and anemone symbiosis Tsikliras AC, éditeur. PLoS ONE. 2014;9(5):e98449. 24878777 10.1371/journal.pone.0098449 PMC4039484

[49] Karplus I. The associations between fishes and molluscs. In: Symbiosis in fishes. 2014. p. 230‑75. 10.1002/9781118759769.ch6.

[50] Randall J, Fautin D. Fishes other than anemonefishes that associate with sea anemones. Coral Reefs. 2002;21(2):188–90.

[51] Gusmão LC, Daly M. Evolution of sea anemones (Cnidaria: Actiniaria: Hormathiidae) symbiotic with hermit crabs. Mol Phylogenet Evol. 2010;56(3):868–77. 20457262 10.1016/j.ympev.2010.05.001

[52] Greenwood PG, Garry K, Hunter A, Jennings M. Adaptable defense: a nudibranch mucus inhibits nematocyst discharge and changes with prey type. Biol Bull. 2004;206(2):113–20. 15111366 10.2307/1543542

[53] Roux N, Logeux V, Trouillard N, Pillot R, Magré K, Salis P, et al. A star is born again: methods for larval rearing of an emerging model organism, the false clownfish Amphiprion ocellaris. J Exp Zoolog B Mol Dev Evol. 2021;336(4):376–85. 10.1002/jez.b.23028 PMC8248105 33539680

[54] Salis P, Roux N, Huang D, Marcionetti A, Mouginot P, Reynaud M, et al. Thyroid hormones regulate the formation and environmental plasticity of white bars in clownfishes. Proc Natl Acad Sci. 2021;118(23):e2101634118. 34031155 10.1073/pnas.2101634118 PMC8201804

[55] Salis P, Lorin T, Lewis V, Rey C, Marcionetti A, Escande ML, et al. Developmental and comparative transcriptomic identification of iridophore contribution to white barring in clownfish. GenBank. 2018. https://www.ncbi.nlm.nih.gov/bioproject/PRJNA482393/. 10.1111/pcmr.12766 PMC6483885 30633441

[56] RStudio Team. RStudio: integrated development for R. RStudio, PBC,. Boston, MA UR; 2020.

