# Supplementary figures and images for: Anemonefish use sialic acid metabolism as Trojan horse to avoid giant sea anemone stinging
Source: BMC Biol. 2025 Feb 15;23:39. doi: 10.1186/s12915-025-02144-8 (PMC11829568; doi:10.1186/s12915-025-02144-8)

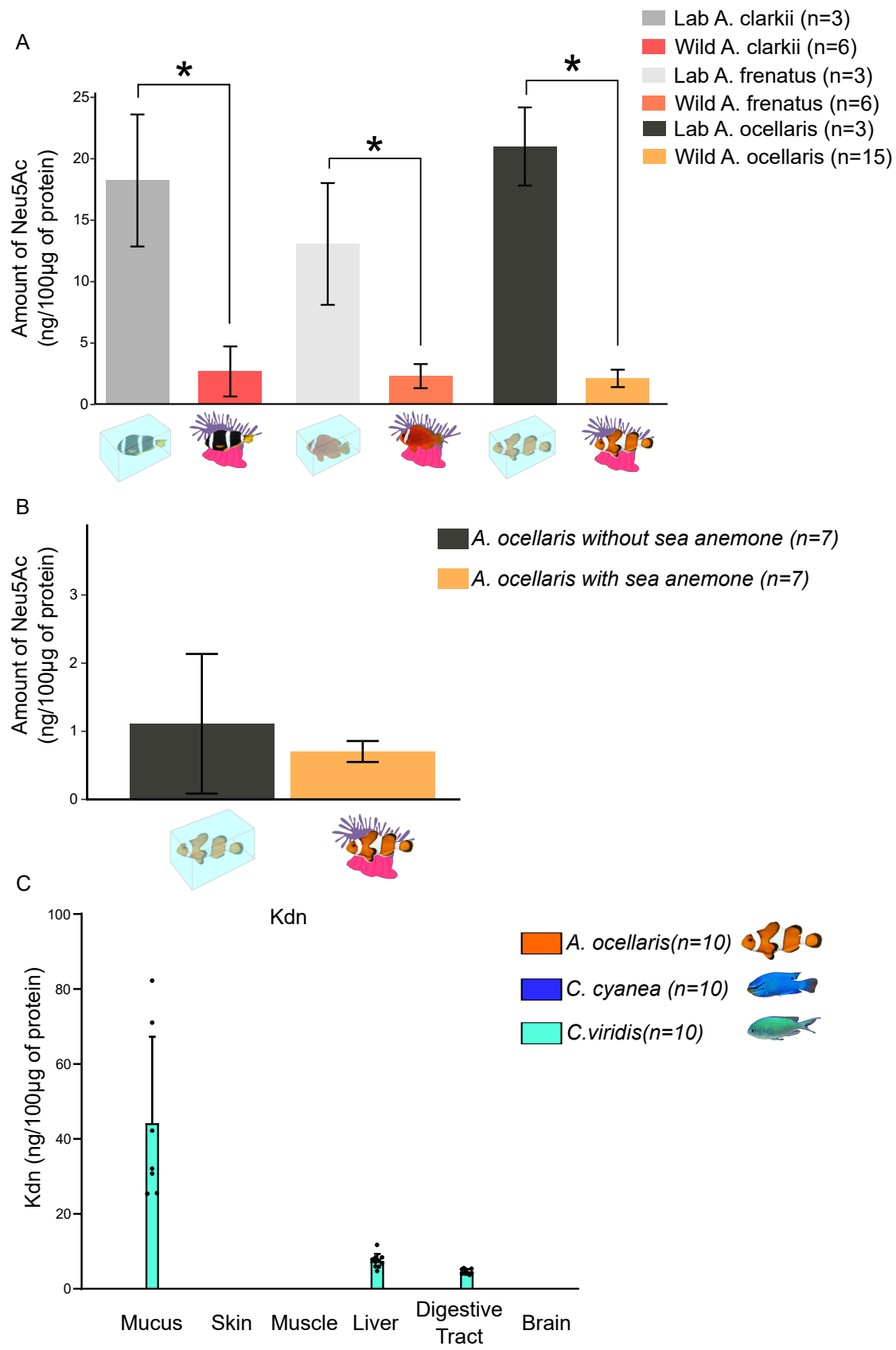

Supplement: Supplementary file 1 — Additional file 1: Fig. S1 A) Comparison of Neu5Ac levels between lab maintained anemonefish species and wild caught anemonefish species (Amphiprion clarkii, Amphiprion frenatus, Amphiprion ocellaris). A parametric test of Student or a non-parametric Wilcoxon-Mann–Whitney test was performed to compare the mean Neu5Ac levels between lab and wild environment for each species. Significant differences are displayed by a star (*) for each species. B) Comparison of Neu5Ac levels between A. ocellaris held without and with sea anemone (S. gigantea) in aquarium at Okinawa marine station. A non-parametric Wilcoxon-Mann–Whitney test was performed but no significant difference was observed between the two groups. C) Levels of Kdn detected in organs of Amphiprion ocellaris, Chrysiptera cyanea, and Chromis viridis. Data are presented in Additional file 6: Table S1. [file 12915_2025_2144_MOESM1_ESM.pdf]

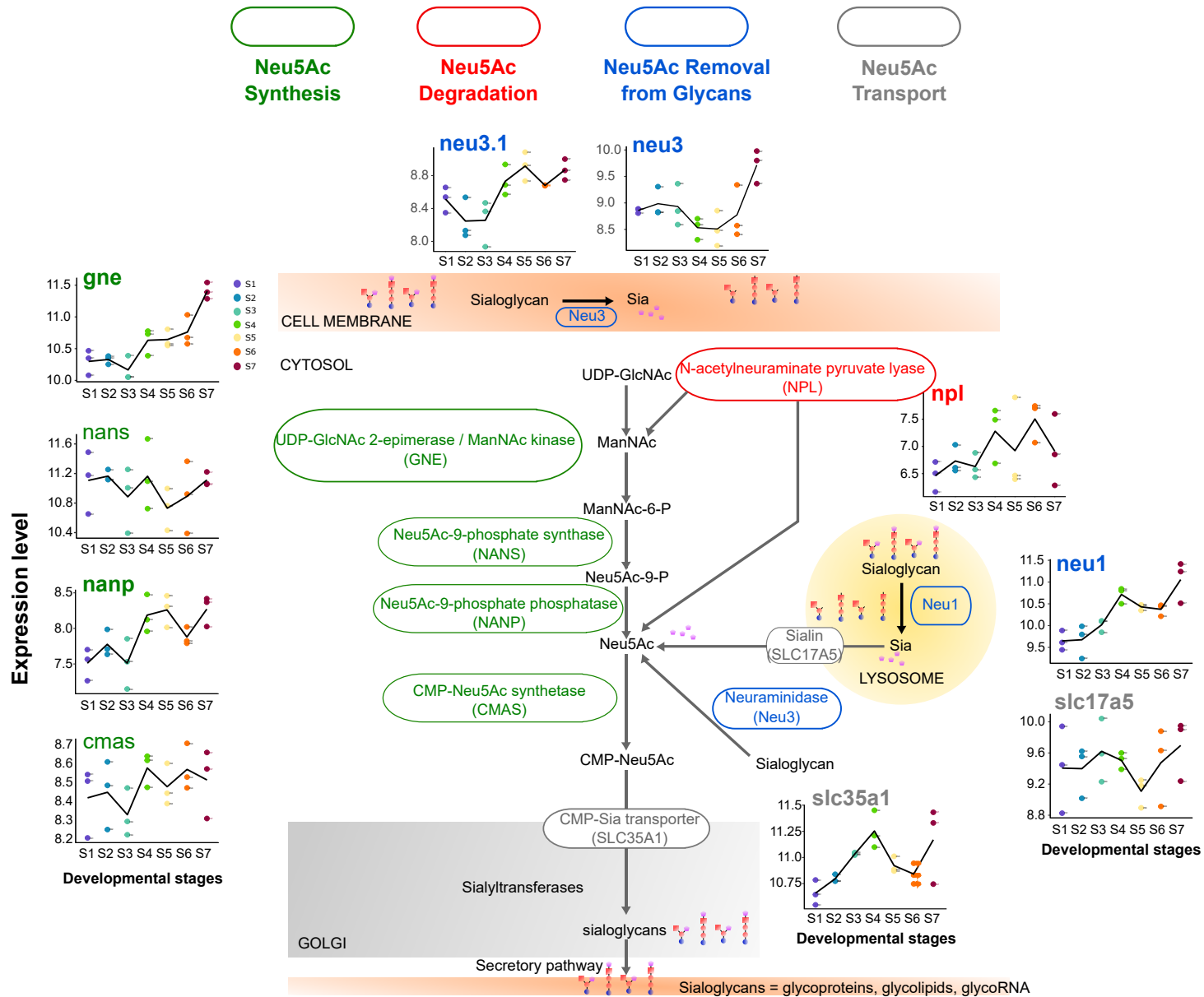

Supplement: Supplementary file 2 — Additional file 2: Fig. S2 Complete signaling pathway of Neu5Ac metabolism associated with gene expression levels retrieved from transcriptomic data obtained by Roux et al. and Salis et al. [37, 54, 55]. Green genes are involved Neu5Ac synthesis, red genes in Neu5Ac degradation, blue genes in Neu5Ac removal from sialoglycans, and gray genes in Neu5Ac transport. Genes written in bold are significantly differentially expressed between pre metamorphosis stage (S1 and/or S2 and/or S3) and metamorphosis stages (S5 and/or S6 and/or S7) (see Roux et al. [1] for the description of gene expression analysis method). Data are presented in Additional file 6: Table S4. [file 12915_2025_2144_MOESM2_ESM.pdf]

Increase

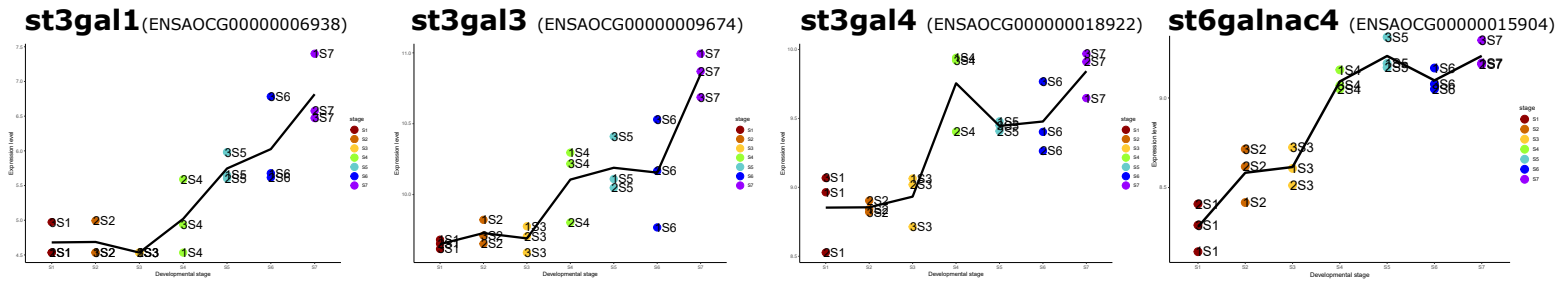

Surge at S4/S5 and surge at S7

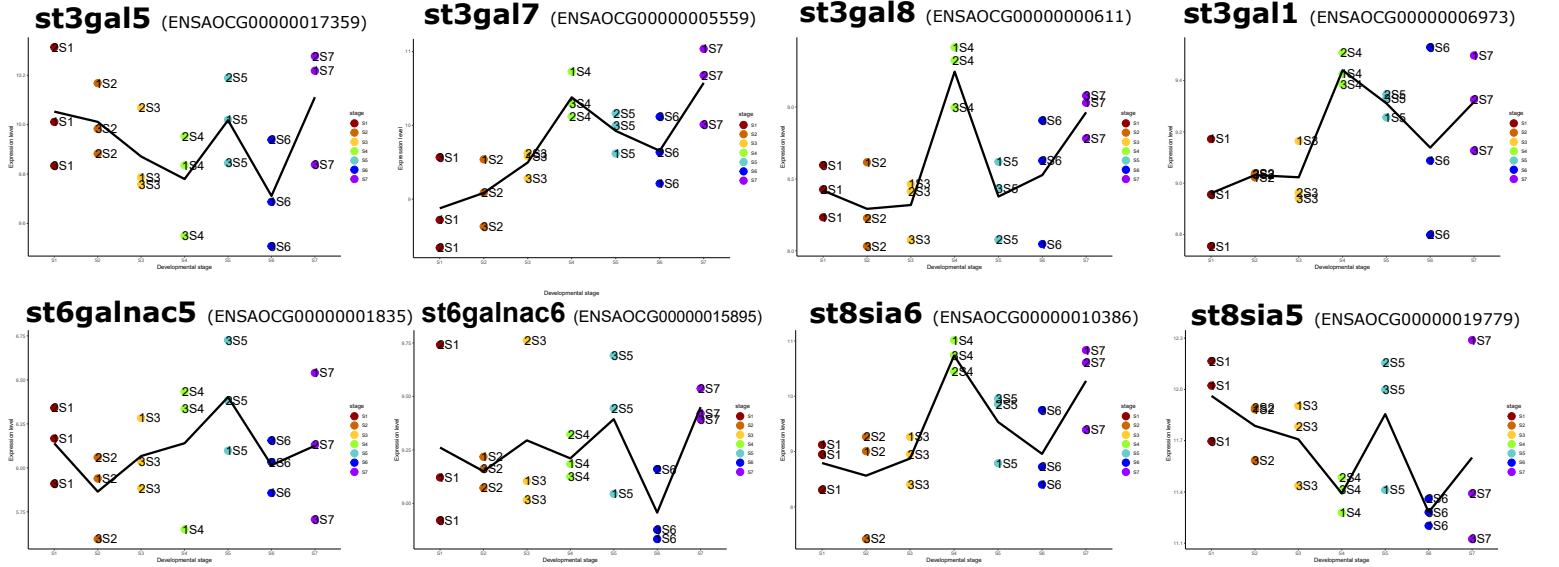

Decrease

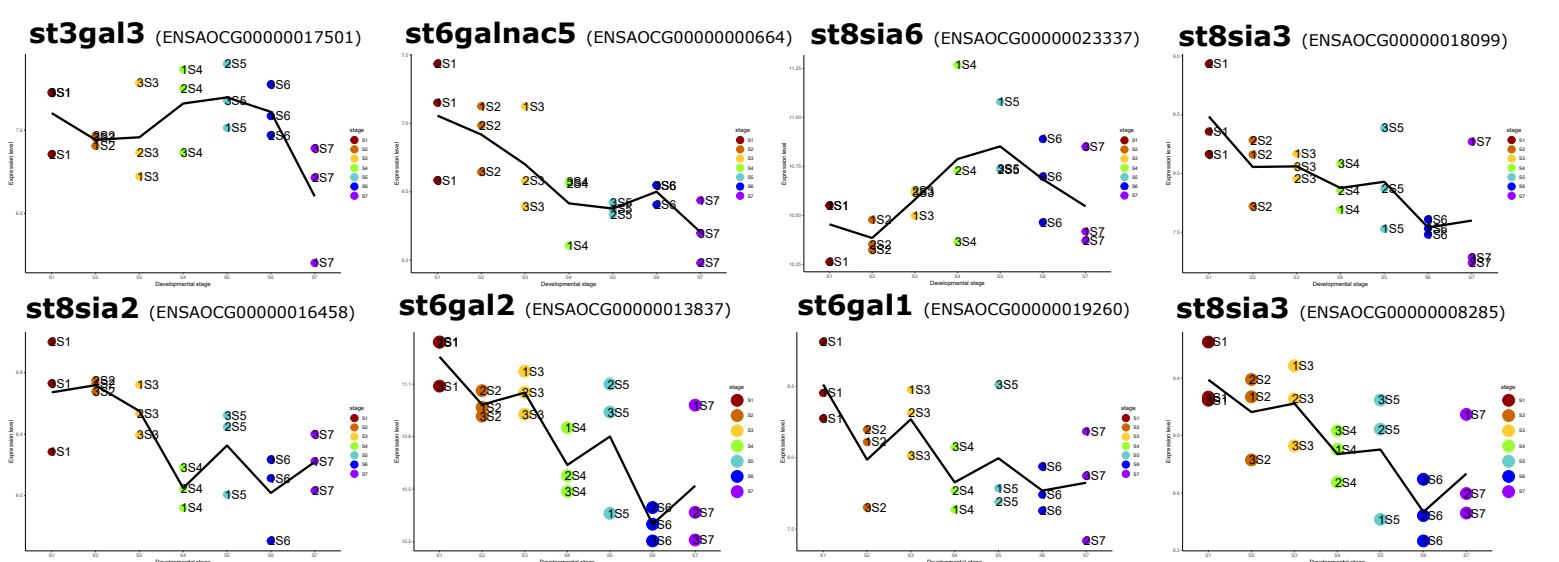

Relatively stable

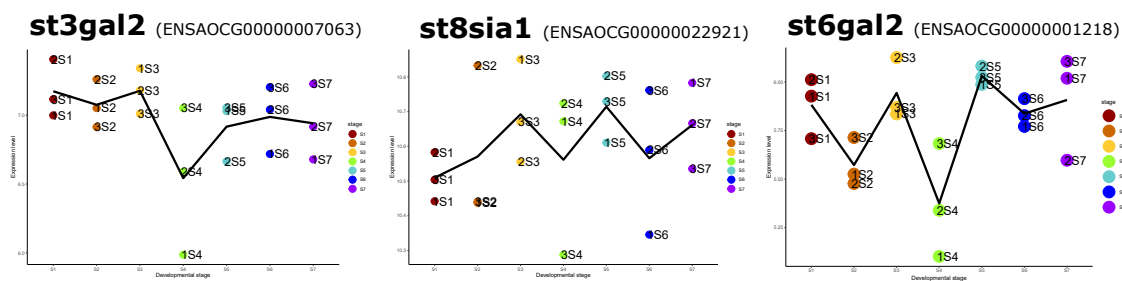

Supplement: Supplementary file 3 — Additional file 3: Fig. S3 Expression levels of genes encoding enzymes, called sialyltransferase, involved in the transfer and fixation of Neu5Ac on sialoglycans. Expression levels were classified into 4 categories (increase, surge at stage 4 or 5, decrease, and relatively stable). Data are presented in Additional file 6: Table S4. [file 12915_2025_2144_MOESM3_ESM.pdf]

Additional file 4, Figure S4 Roux et al

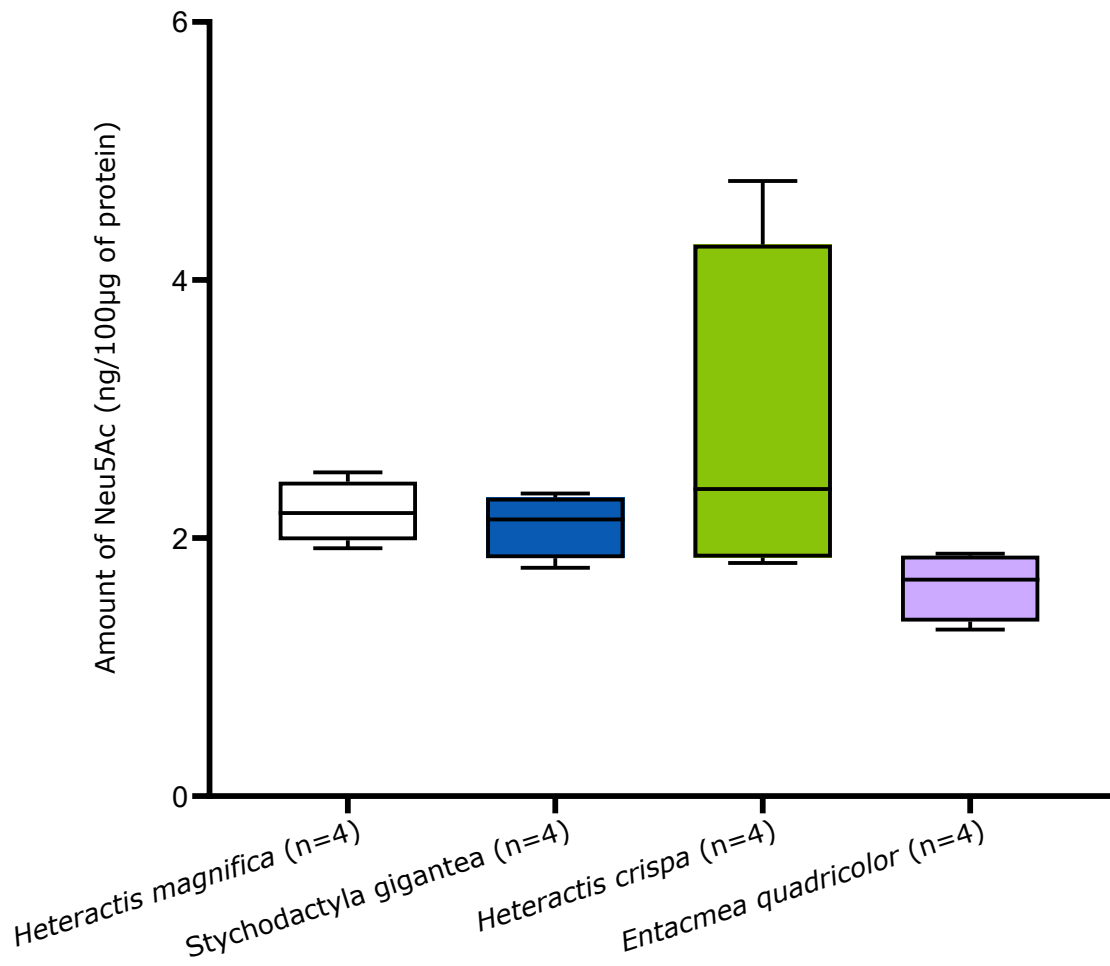

Supplement: Supplementary file 4 — Additional file 4: Fig. S4 Neu5Ac levels in Heteractis magnifica, Stichodactyla gigantea, Heteractis crispa, and Entacmaea quadricolor below the detection limit. Data are presented in Additional file 6: Table S5. [file 12915_2025_2144_MOESM4_ESM.pdf]

Additional file 5, Figure S5 Roux et al.,

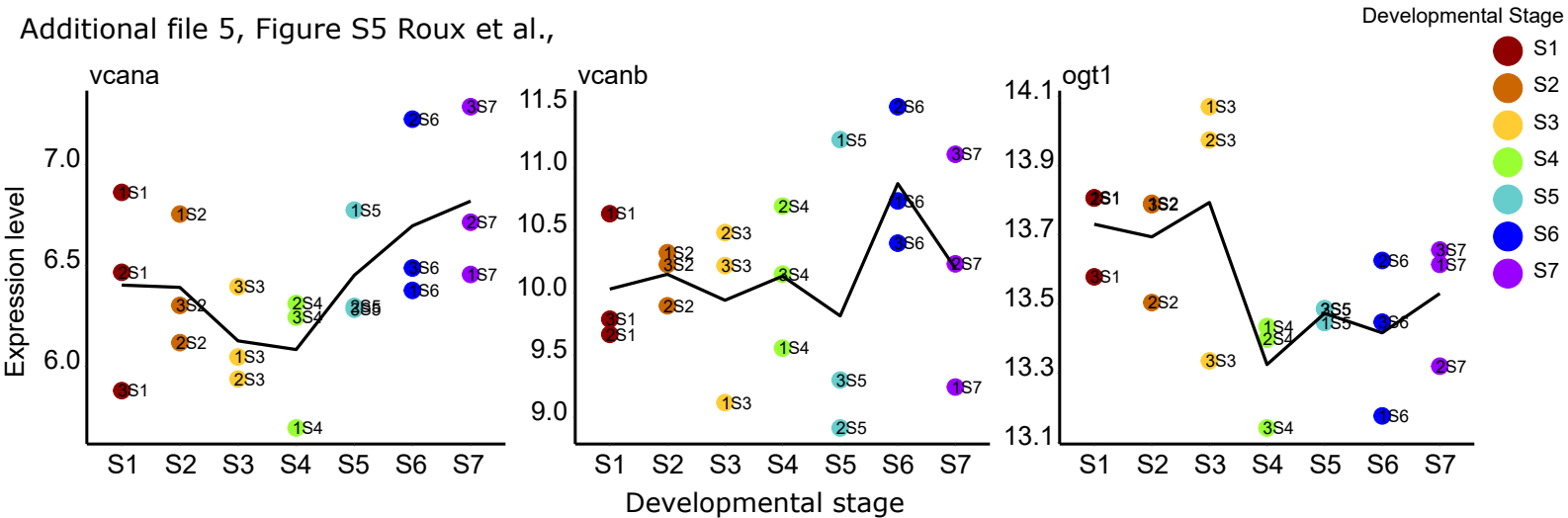

Supplement: Supplementary file 5 — Additional file 5: Fig. S5 Expression levels of genes encoding proteins with a functional link with N-acetylated sugars: versican core protein (vcan) and the O-GlcNAc transferase (ogt) [22]. Versican core protein is known to be a critical extracellular matrix regulator of immunity and inflammation [43] that interacts with several matrix molecules including glycosaminoglycans containing N-acetylhexosamine [44] (see Roux et al. [37] for the description of gene expression analysis method). Data are presented in Additional file 6: Table S4. [file 12915_2025_2144_MOESM5_ESM.pdf]
